# Autism in Criminal-Justice and Forensic Settings: A Systematic Review and Meta-Analysis with an Exploratory Bidirectional Synthesis

**DOI:** 10.3390/bs16101923

**Published:** 2026-10-16

**Authors:** Risvan Vlad Rusu, Cristian Delcea

**Affiliations:** 1 Department of Research, Institute of Sexology, 400394 Cluj-Napoca, Romania; risvanvladrusu@sexology.ro; 2 Multidisciplinary Doctoral School, Vasile Goldiș Western University of Arad, 310025 Arad, Romania

**Keywords:** autism, criminal justice, criminal-justice involvement, forensic psychiatry, prison, prevalence, offending, neurodevelopmental disorders

## Abstract

Background: Estimates of autism in criminal-justice and forensic populations vary widely, while studies involving autism-defined populations report heterogeneous justice outcomes. Broad pooling can obscure differences in recruitment, diagnostic ascertainment, legal context, and outcome definition. Methods: We conducted a systematic review and meta-analysis addressing two complementary questions: the proportion of autistic people in defined criminal-justice and forensic populations (Axis A) and criminal-justice outcomes within autism-defined populations (Axis B). Searches covered PubMed/MEDLINE, Lens.org, NCJRS, Scopus, Web of Science Core Collection, and ProQuest Central Premium, supplemented by citation searching. Compatible exact-count proportions were synthesized using binomial generalized linear mixed models with profile-likelihood confidence intervals; inverse-variance REML models with modified Hartung–Knapp inference were used for sensitivity analyses. Results: The reconciled register contained 300 candidate reports, 161 retained reports, and 37 selected numerical contributions from 30 reports representing 29 source-study units. Only three clinically compatible Axis A strata supported pooling. Estimated proportions of autism were 23.57% (95% CI 10.67–42.69) in specialist forensic intellectual-disability services, 0.89% (0.36–2.23) in general forensic responsibility evaluations, and 23.51% (20.10–27.08) in forensic youth referral services. Each synthesis contained two cohorts, and modified Hartung–Knapp intervals were substantially wider. General-prison administrative estimates ranged from 0.62% to 1.09%. Axis B findings differed by endpoint, population, sex, observation period, and adjustment set and were therefore not pooled. Conclusions: Autism frequency in justice and forensic settings is strongly setting- and ascertainment-dependent. The evidence does not support a single criminal-justice prevalence estimate or a unitary association between autism and offending. Clinically and legally defined populations should be synthesized separately, with explicit management of cohort overlap and diagnostic definitions.

## 1. Introduction

### 1.1. Autism and Criminal-Justice Involvement: An Inconsistent Evidence Base

Autism is a heterogeneous neurodevelopmental condition characterized by persistent differences in social communication and social interaction, together with restricted or repetitive patterns of behavior, interests, activities, or sensory processing. These characteristics vary considerably between individuals and across developmental stages, and their functional implications depend strongly on intellectual ability, language, co-occurring psychiatric and neurodevelopmental conditions, environmental demands, and the availability of appropriate support. Consequently, the relevance of autism to criminal-justice and forensic settings cannot be reduced to a simple question of whether autistic people are more or less likely to offend. Contact with police, courts, correctional institutions, probation services, and forensic mental-health systems occurs within complex social and institutional contexts in which communication demands, sensory conditions, diagnostic recognition, vulnerability, and access to accommodations may substantially affect both experiences and outcomes (Collins et al., 2023; Cooper et al., 2022).

This distinction is important because criminal-justice involvement encompasses substantially different processes. Police contact may occur because an autistic person is suspected of an offense, but it may also arise in the context of victimization, vulnerability, behavioral crisis, safeguarding concerns, or misunderstanding. Arrest, charge, prosecution, conviction, imprisonment, forensic psychiatric assessment, and admission to secure mental-health services likewise represent different stages and different selection mechanisms. Treating these experiences as interchangeable risks collapsing heterogeneous pathways into a single construct of “criminality.” Previous reviews have therefore emphasized both the vulnerability of autistic people during criminal-justice encounters and the methodological difficulty of drawing broad conclusions about offending from highly selected forensic samples (Collins et al., 2023; Cooper et al., 2022; King & Murphy, 2014).

The characteristics associated with autism may also become particularly consequential once an individual encounters the criminal-justice system. Communication differences can complicate interactions in police custody and formal interviews, while unfamiliar routines, heightened anxiety, sensory demands, and difficulties interpreting ambiguous social situations may interfere with effective participation. Qualitative and participatory research on police custody has, for example, identified communication barriers, anxiety, sensory difficulties, and environmental demands as important problems for autistic detainees, while police personnel have reported difficulties in recognizing autism and providing appropriate support within the constraints of custody procedures (Holloway et al., 2020). These issues reinforce the need to distinguish between the prevalence of autism within justice settings, the experiences of autistic people within those settings, and the probability of criminal behavior itself. These are related questions, but they are not equivalent.

### 1.2. Heterogeneity Across Criminal-Justice and Forensic Settings

Research on autism and the criminal-justice system has developed considerably during the past two decades, but estimates of both autism within justice populations and justice involvement among autistic populations remain strikingly heterogeneous. King and Murphy (2014), in an influential early systematic review, highlighted substantial methodological limitations in the available literature and cautioned against simple conclusions concerning an association between autism and offending. Subsequent research expanded rapidly, prompting Collins et al. (2023) to update the evidence published after that earlier review. Their systematic review identified 47 papers and concluded that, despite the growth of the literature, relatively little high-quality research had resolved the methodological problems identified previously. In particular, methodological differences continued to complicate direct comparisons between autistic and non-autistic populations (Collins et al., 2023).

A broader systematic review of autism and criminal-justice intersections similarly identified evidence distributed across multiple stages of the justice process rather than a single homogeneous outcome. Cooper et al. (2022) organized 89 articles according to stages of the criminal-justice system and emphasized that social and communication differences may affect interactions with justice institutions and that evidence-based policy and practice responses remain necessary across these stages. Taken together, these reviews suggest that the major conceptual problem is not merely that estimates differ between studies. Rather, studies often investigate fundamentally different populations, processes, and outcomes while placing them under the same broad label of criminal-justice involvement.

The most recent comprehensive synthesis by Chester et al. (2025) made this heterogeneity particularly visible. Their systematic review included 41 studies, of which 25 examined autism prevalence within criminal-justice or forensic cohorts and 16 examined criminal-justice or forensic involvement among autistic populations. Autism estimates within justice and forensic populations ranged from 0% to 60%, whereas reported justice or forensic involvement among autistic populations ranged from 3% to 71% (Chester et al., 2025). The breadth of these ranges is informative, but it also illustrates why the literature cannot be interpreted as though all studies were estimating the same underlying parameter.

Chester et al. (2025) further observed that most studies examining autism within criminal-justice or forensic populations reported proportions exceeding the approximately 1% general-population benchmark used in that review, whereas studies beginning with autistic cohorts tended to indicate offending at lower or broadly comparable levels relative to comparison groups. At first sight, these two observations may appear contradictory. They are not necessarily so. They arise from opposite sampling directions and therefore answer different epidemiological questions. A study asking how many patients in a specialist forensic service are autistic conditions on entry into that service. A population cohort asking whether autistic people subsequently receive criminal convictions begins with autism status and observes a later justice outcome. The denominators, selection mechanisms, and target parameters are fundamentally different.

This bidirectional distinction is central to interpretation. High autism proportions in a specialized forensic population do not establish that autistic people in the general population have an equivalently elevated probability of offending. Conversely, a low rate of recorded autism in a prison register does not necessarily establish a low prevalence of autism among incarcerated people, because administrative diagnostic coding depends on previous recognition, access to assessment, coding practices, and the completeness of institutional records. The direction of sampling therefore needs to be made explicit before results from different studies can meaningfully be compared.

A second major difficulty is that the “criminal justice system” is not a single population or sampling frame. Police contacts, community justice services, prisons, courts, forensic psychiatric assessments, secure hospitals, forensic intellectual-disabilityservices, and specialist youth services select people for very different reasons. Even within ostensibly similar settings, referral criteria may differ substantially between jurisdictions and institutions. A national prison register attempts to describe a broad correctional population, whereas a secure forensic intellectual-disability service is deliberately enriched for complex neurodevelopmental, psychiatric, behavioral, and legal needs. A forensic responsibility evaluation population is selected because a legal question has prompted psychiatric assessment. A specialist youth forensic service receives referrals according to clinical and legal criteria that differ from those determining entry into either a prison or a general community sample.

This distinction has direct consequences for prevalence estimation. In the evidence synthesized in the present review, for example, administrative prison-register studies, specialist forensic intellectual-disability services, general forensic responsibility evaluations, offense-selected custodial samples, and forensic youth referral services represent recognizably different populations. Combining them simply because each has some connection to the justice system would imply a common target population that does not exist. A pooled estimate might therefore achieve statistical precision while losing epidemiological and clinical meaning.

Previous systematic reviews have encountered precisely this problem. Chester et al. (2025) explicitly distinguished criminal-justice settings involving police, courts, and prisons from forensic psychiatric services and forensic psychiatric assessment populations. Collins et al. (2023) likewise emphasized continuing methodological limitations and difficulties comparing samples across the literature. The persistent heterogeneity documented across successive reviews suggests that it should not be treated solely as statistical noise around a hypothetical common prevalence. At least part of that heterogeneity is structural; different studies sample different populations.

Intellectual disability is particularly important in this respect. Autism and intellectual disability frequently co-occur, and specialist forensic intellectual-disability services are selected around clinical complexity. Estimates from these services consequently address the composition of specialist clinical caseloads rather than the prevalence of autism in an unselected criminal-justice population. Similar concerns apply to offense-selected samples, such as populations recruited specifically because of violent or sexual offending. Such samples may be clinically informative, but their selection criteria prevent straightforward extrapolation to general prison populations or to autistic people in the community.

Age and developmental context further complicate comparisons. Youth justice and forensic child and adolescent services operate within legal and developmental frameworks that differ from adult criminal-justice systems. Diagnostic recognition may also differ by age, particularly where young people enter specialist services because of behavioral, educational, psychiatric, or competency concerns. Combining youth referral populations with adult prison or forensic psychiatric populations may therefore obscure rather than explain variation.

### 1.3. Diagnostic Ascertainment as a Source of Heterogeneity

Variation in autism ascertainment constitutes another major source of between-study differences. Across the literature, autism may be identified through administrative diagnostic codes, routine clinical records, structured specialist assessment, screening instruments, retrospective chart review, self-report, or historical diagnostic categories. These approaches do not have equivalent sensitivity, specificity, or implications for prevalence estimation.

Administrative registers are particularly useful for large population-based studies because they allow very large samples and longitudinal linkage. However, they usually identify recorded diagnoses rather than previously unrecognized autism. The resulting estimate is therefore more accurately interpreted as the proportion of individuals with autism documented in the relevant register, rather than the prevalence that would have been obtained through systematic diagnostic assessment. Conversely, studies using active specialist assessment may identify previously unrecognized cases but are often conducted in much smaller or more selected samples.

Historical diagnostic terminology creates additional problems. Studies spanning several decades may report autistic disorder, Asperger syndrome, pervasive developmental disorder, or broader diagnostic groupings that do not correspond directly to contemporary autism spectrum disorder. Treating all such categories as interchangeable can artificially increase apparent comparability between studies. At the same time, excluding all historical studies would discard potentially informative longitudinal evidence. A defensible synthesis therefore needs to retain original diagnostic definitions and distinguish historical or uncertain categories from contemporary confirmed autism rather than silently harmonizing them.

Screening-positive status also requires separation from diagnosis. Screening instruments can identify individuals who may warrant diagnostic assessment, but a threshold score is not equivalent to a clinical diagnosis. Similarly, suspected autism recorded by clinicians or services represents a different evidentiary category from confirmed autism. Combining confirmed diagnoses, suspected cases, and screening-positive individuals in a single numerator can substantially alter estimates and make comparisons across studies misleading.

These distinctions are especially important in forensic populations, where opportunities for diagnostic assessment may themselves be associated with service pathways. Individuals in specialist psychiatric or intellectual-disability services may undergo more extensive assessment than those in general prisons or community justice settings. Higher observed autism proportions may therefore reflect both genuine differences in population composition and greater diagnostic opportunity. Conversely, under-recognition in general correctional populations may produce artificially low recorded-diagnosis estimates. The observed proportion is thus partly a function of who enters the setting and partly a function of how autism is sought and documented.

### 1.4. The Reverse Direction: Justice Outcomes in Autism-Defined Populations

The literature beginning with autism-defined cohorts poses a different set of methodological problems. Here, the relevant question is not how frequently autism occurs within a justice setting, but what proportion of autistic people experience particular justice outcomes, or whether such outcomes differ relative to comparison populations. This direction is crucial because conclusions drawn from forensic samples cannot simply be reversed to characterize autistic people generally.

Even within this literature, however, “criminal-justice involvement” encompasses heterogeneous outcomes. Police interaction may occur without arrest or offending. Arrest is not equivalent to charge; charge is not equivalent to conviction; conviction is not equivalent to violent offending; and reconviction after forensic psychiatric treatment is conditional on an individual already having entered a highly selected forensic pathway. Self-reported illegal behavior, administrative criminal records, court records, and registry-defined convictions also differ in ascertainment.

Consequently, a single pooled “offending rate” may obscure more than it clarifies. Collins et al. (2023) concluded that methodological limitations continued to make comparisons between autistic and non-autistic offenders difficult, despite the expansion of the evidence base. Chester et al. (2025) similarly found wide variation in reported criminal-justice or forensic involvement among autistic cohorts and noted that comparative studies generally did not provide a simple picture of increased offending among autistic people. These findings argue for outcome-specific synthesis rather than assuming that all justice contacts index the same underlying behavior.

Temporal ordering is equally important. A cohort defined by autism before subsequent justice outcomes provides a different basis for inference from a forensic sample in which autism is identified after an offense or during court-ordered assessment. Retrospective lifetime histories may establish that both autism and justice involvement occurred, but they may not always establish the temporal relationship required for interpreting autism as preceding the measured outcome. The present synthesis therefore distinguishes prospective or temporally interpretable autism-defined cohorts from evidence conditional on prior justice involvement.

Effect measures also complicate synthesis. Studies report simple proportions, risk ratios, odds ratios, hazard ratios, incidence-rate ratios, and survival estimates. These measures are not interchangeable, particularly when follow-up periods differ. An odds ratio for violent conviction, a hazard ratio for time to reconviction, and the percentage of participants reporting police contact cannot legitimately be combined merely because all concern criminal-justice outcomes.

Adjustment strategies introduce further interpretive complexity. Neurodevelopmental and psychiatric co-occurrence is common, and variables such as attention-deficit/hyperactivity disorder, conduct disorder, intellectual disability, substance use, and psychiatric morbidity may be associated with both autism and justice outcomes. However, adjustment is not automatically preferable simply because more covariates are included. Depending on the causal structure, a covariate may function as a confounder, mediator, collider, or marker of selection. Studies using different adjustment sets may therefore estimate different conditional associations even when they use the same underlying population.

### 1.5. Cohort Dependence and the Problem of Apparent Replication

The growth of large administrative and population registers has substantially strengthened autism research, but it has also created a less visible problem for evidence synthesis; multiple publications may analyze overlapping populations. National registers can generate several papers addressing different outcomes, age ranges, follow-up periods, diagnostic definitions, or statistical models. Likewise, specialist clinical services may publish repeated analyses of partially overlapping cohorts.

If each publication is treated as an independent study, participants may effectively be counted multiple times. This pseudo-replication can inflate the apparent size of the evidence base, distort weighting in meta-analysis, and produce misleading impressions of consistency. Publication counts, effect estimates, source studies, and independent participant cohorts therefore need to be distinguished explicitly.

The issue becomes particularly important when sex-specific estimates or alternative statistical models are reported. Male and female estimates derived from a single national cohort are informative subgroup results, but they are not independent replications. Similarly, several adjusted models reported for the same outcome should not enter a meta-analysis as though they represented separate samples. A synthesis must therefore map reports to source studies and participant cohorts before deciding which estimates can legitimately be combined.

This problem has received less attention than conventional statistical heterogeneity, yet it is fundamental to the validity of meta-analysis in register-rich fields. Increasing the number of effect sizes does not increase the amount of independent information when those effects repeatedly describe the same participants. A smaller synthesis of clearly independent and compatible cohorts may therefore provide a more defensible estimate than a larger meta-analysis assembled from overlapping reports.

### 1.6. Why a Stratified Meta-Analytic Approach Is Needed

The existing review literature has established several findings with reasonable consistency. First, autistic people are represented across police, court, correctional, and forensic psychiatric settings. Second, reported proportions vary dramatically between settings. Third, justice outcomes among autistic cohorts are heterogeneous and do not support a simple characterization of autism as uniformly associated with greater offending. Fourth, methodological quality and comparability remain persistent concerns (Chester et al., 2025; Collins et al., 2023; Cooper et al., 2022; King & Murphy, 2014).

What remains less well resolved is how much of the apparent variation can meaningfully be synthesized quantitatively without sacrificing interpretability. Chester et al. (2025) synthesized 41 studies narratively and documented autism estimates ranging from 0% to 60% in justice or forensic settings and justice involvement ranging from 3% to 71% among autistic populations. These ranges demonstrate the breadth of the evidence but do not provide population-specific pooled estimates. Conversely, indiscriminate meta-analysis across all available settings would risk answering a question that has no coherent denominator.

The key issue is therefore not whether meta-analysis is possible in a purely computational sense, but whether the studies entering a particular model estimate sufficiently similar quantities. Pooling should depend on substantive compatibility in recruitment frame, represented population, autism ascertainment, outcome definition, observation period, and unit of analysis. Statistical similarity alone cannot repair incompatible sampling frames. Conversely, a small number of clinically compatible studies may justify a narrowly defined synthesis even when the resulting uncertainty is substantial.

Sparse meta-analysis introduces its own challenges. Random-effects models based on very few studies provide limited information about between-study variance, and conventional large-sample inference may convey misleading precision. Alternative modeling approaches and small-sample sensitivity analyses are therefore particularly important when only two or three compatible cohorts are available. Generalized linear mixed models (GLMMs) offer an attractive approach for proportions because they model binomial data directly and avoid some problems associated with transformed proportions and continuity corrections (Lin & Chu, 2020). At the same time, sensitivity to inferential method remains important, particularly when the number of studies is very small. Modified Hartung–Knapp procedures provide one means of illustrating the degree to which uncertainty can expand when small-sample inference is acknowledged (Röver et al., 2015).

The methodological objective should consequently be neither maximal pooling nor maximal fragmentation. It should be to identify the narrowest defensible strata that retain clinical meaning while providing enough independent information for quantitative synthesis. Where this condition is not met, individual estimates and structured narrative synthesis are preferable to a pooled number whose interpretation is unclear.

### 1.7. The Present Study

Against this background, the present systematic review and meta-analysis adopts a deliberately stratified and bidirectional framework. Rather than treating criminal-justice and forensic populations as a single sampling universe, the review distinguishes correctional populations, specialist forensic services, forensic assessment populations, and youth referral pathways according to how individuals entered the sample and how autism was ascertained. Historical diagnostic categories, administrative diagnoses, routine clinical documentation, structured assessments, screening-positive status, and suspected autism are not automatically treated as equivalent.

The primary meta-analytic question, Axis A, concerns the proportion of autistic individuals within defined criminal-justice and forensic populations. Quantitative pooling is restricted to strata in which recruitment frame, represented population, diagnostic ascertainment, denominator, and unit of analysis are sufficiently compatible. The aim is therefore not to derive an overall prevalence of autism “in the criminal justice system,” because no single criminal-justice sampling frame exists. Instead, the analysis seeks population-specific estimates whose denominators retain clinical and legal meaning.

The complementary Axis B refers to autism-defined populations and examines criminal-justice outcomes. This axis preserves distinctions between police interaction, arrest or charge, conviction, violent outcomes, cyber offending, intimate-partner-violence arrest, and reconviction. Observation periods, effect metrics, adjustment sets, and cohort dependence are retained rather than harmonized artificially. Where independent and sufficiently comparable evidence is unavailable, results are presented individually rather than forced into a common pooled effect.

This approach also explicitly addresses dependence between publications and cohorts. Reports are mapped to underlying source studies and participant populations, and overlapping samples, alternative models, repeated observations, and subgroup estimates are not treated as independent evidence within the same synthesis. This distinction is particularly important for registry studies and repeatedly reported specialist-service cohorts.

Accordingly, the present review has four objectives, as follows:

1. To identify and classify quantitative evidence on autism across correctional, forensic-service, forensic-evaluation, court, and youth-referral populations;
2. To estimate autism proportions only within clinically and methodologically compatible Axis A strata using models appropriate for binomial proportion data;
3. To evaluate the robustness of pooled estimates to small-sample inference, alternative source-supported definitions, and cohort-selection decisions; and
4. To characterize Axis B justice outcomes without combining conceptually different endpoints or statistically dependent samples.

## 2. Methods

### 2.1. Design and Reporting Framework

**Figure 1:**
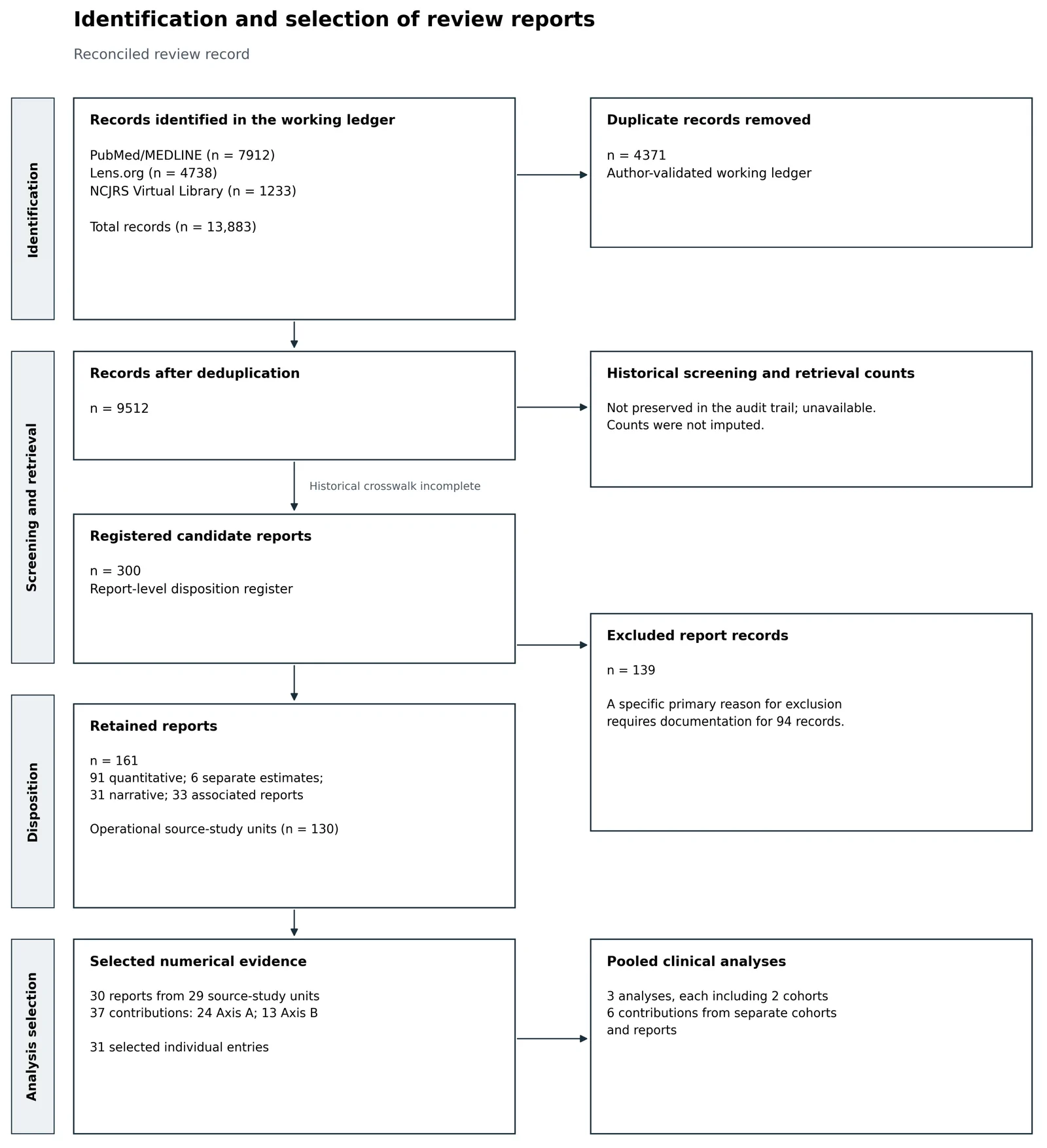
PRISMA 2020 study-selection flow diagram for the reconciled review record. Historical intermediate screening counts that were not preserved in the audit trail are explicitly identified rather than imputed.

This systematic review and meta-analysis was conducted in accordance with the Preferred Reporting Items for Systematic Reviews and Meta-Analyses (PRISMA 2020) statement (Page et al., 2021), with search reporting informed by PRISMA-S (Rethlefsen et al., 2021). A completed PRISMA 2020 checklist is provided in the Supplementary Materials (PRISMA_2020_Checklist.docx). The complete study selection process is illustrated in the PRISMA 2020 flow diagram presented in Figure 1. The review addresses two complementary questions: the proportion of autistic people in defined criminal justice and forensic populations (Axis A) and criminal justice outcomes within autism-defined populations (Axis B). Axis A is the primary synthesis; Axis B provides an exploratory mapping of outcome-specific evidence.

The review protocol was not prospectively registered (e.g., in PROSPERO). The initial searches and analyses preceded the revised synthesis framework. Additional searches were conducted in September 2026, and the revision plan was documented on 23–29 September 2026, after the evidence had been examined. Changes included narrower population and diagnostic strata, explicit rules for cohort overlap, removal of overall Axis A and broad B1 pooling, and revised statistical methods. These are revision-stage amendments rather than prospectively specified decisions. Supplementary Methods SM2 documents their chronology and rationale. The analyses and reassessments specified below constitute the revised analysis plan and were completed before the revised results were finalized.

### 2.2. Eligibility Criteria

Eligible evidence comprised empirical studies of participants of any age in which autism and criminal justice involvement were operationally defined. Cross-sectional surveys, cohorts, registry studies, and analytic case-control studies were eligible for the questions supported by their designs. Axis A required a defined justice or forensic recruitment frame and a denominator corresponding to that frame. A bounded clinical series could inform an estimate for a selected service but could not establish prevalence in the wider correctional population. Mixed civil and criminal samples required an identifiable eligible criminal justice subset for quantitative synthesis.

Axis B required an autism-defined population and an identifiable justice outcome or comparative association. Autism established before outcome ascertainment was required for estimates interpreted as subsequent outcomes. Retrospective lifetime outcomes with uncertain temporal ordering were retained only as separately labelled descriptive evidence. Case reports, reviews, editorials, qualitative-only reports, and reports without usable quantitative data did not contribute to meta-analysis; relevant material could inform the narrative context. General challenging behavior, self-injury, or aggression without an explicitly defined eligible outcome with regard to violence or justice was not treated as criminal justice involvement. Eligibility for the review was distinguished from eligibility for an individual pooled analysis.

### 2.3. Population and Diagnostic Classification

Axis A was classified by the process through which participants entered the sample. Within correctional populations (A1), general prison samples, police custody, community corrections, offense-selected samples, and clinically selected samples were distinguished. Within forensic services (A2), intellectual-disability services were separated from other secure forensic services, long-stay services, and mixed or otherwise selected services. Within evaluation and referral populations (A3), general forensic evaluations, juvenile pretrial evaluations, offense-selected evaluations, competence assessments, court or liaison and diversion services, and forensic child and adolescent referrals were distinguished. Age-specific and jurisdiction-specific referral pathways were retained when they materially changed the population represented.

Autism ascertainment was classified as specialist or structured diagnostic assessment, routine clinical documentation, or administrative diagnostic coding. These sources define separate primary synthesis strata. Historical autistic disorder and Asperger diagnoses were recorded with their original criteria; undifferentiated pervasive developmental disorder diagnoses were not automatically equated with current autism spectrum disorder. Historical pervasive developmental disorder, screening-positive, suspected-autism, and diagnostically unclear estimates were presented separately from confirmed-diagnosis estimates. Screening instruments were not treated as diagnostic assessments solely because a threshold was reported.

For Axis B, recruitment from an autistic population independently of justice involvement was distinguished from recruitment conditional on custody, forensic referral, court involvement, or an earlier offense. These estimates describe outcomes within a selected population. B1 outcomes were classified separately as police contact, arrest, charge, criminal proceedings, conviction, or reported illegal behavior. The participant’s role in police contact was recorded where available. B2 distinguished violence-specific outcomes, including intimate partner violence. B3 comprised recurrence after a defined index event and required an explicit population at risk. These labels organize the evidence and do not imply that outcomes within each label can be pooled.

### 2.4. Study Selection and Record Management

Records were managed in Rayyan and reconciled in a report-level disposition register. Duplicate records were identified through DOI and bibliographic matching, with title, author, and publication-year information used to resolve ambiguous matches. Multiple publications from the same study were linked rather than treated as duplicate reports. Two reviewers independently screened titles and abstracts and assessed full texts, with disagreements resolved through discussion and consensus. Eligibility decisions from the expanded searches were reconciled against the same criteria before the revised analyses were finalized. One primary reason was assigned where a specific full-text exclusion reason was recoverable from the preserved record.

Separate identifiers were maintained for bibliographic records, full-text reports, source studies, participant cohorts, and analysis contributions. The PRISMA flow distinguishes bibliographic records, report-level dispositions, source-study units, and analysis contributions. A report retained for context or as an associated publication was not counted as an additional independent quantitative study. Where preserved logs did not support an exact historical intermediate count, no count was imputed by subtraction.

### 2.5. Data Collection and Cohort Dependence

For the initial review, one reviewer extracted the data and a second independently checked eligibility, classification, and effect-defining fields. The expanded register records source locations and adjudication notes for revised or disputed entries. Before reanalysis, all variables that determined synthesis membership or effect calculation were checked against the full text and Supplementary Material. Extracted items include country and recruitment dates, study design, recruitment pathway, legal status, age, reported sex or gender, intellectual disability, comorbidity, autism definition and ascertainment, outcome definition and source, observation period, numerator, denominator, missingness, and adjustment variables.

Exact counts were distinguished from rounded percentages, weighted estimates, and model-based results. Rounded percentages were not converted into purportedly exact event counts. A baseline sample size was not substituted for an unreported follow-up denominator. Absence of an Axis I disorder was not coded as missing diagnostic information. If an outcome-specific count, denominator, or diagnostic definition could not be established, the estimate was retained descriptively where interpretable and excluded from exact-count pooling.

Potential overlap was evaluated using recruitment setting, dates, eligibility criteria, sample characteristics, authorship, and explicit statements linking reports. One contribution per independent cohort entered each synthesis. Disjoint cohorts within one publication could contribute separately; parent cohorts and their subsets, repeated observations, and potentially shared participants were not counted as independent within the same model. Primary selection favored the report with the appropriate population coverage, clearest measurement, and most complete eligible denominator. Uncertain overlap was handled conservatively through alternative selections. Decisions were recorded in Supplementary Table S5 without selecting estimates on the basis of their magnitude or statistical significance.

### 2.6. Risk-of-Bias Assessment

*Risk of bias was appraised at the level of the specific study component contributing evidence. Axis A prevalence estimates were assessed with the nine-item JBI critical appraisal checklist for prevalence studies (Munn et al., 2015). Axis B longitudinal and registry-based cohort evidence was assessed with the revised JBI cohort tool (Barker et al., 2025), and analytical cross-sectional evidence with the JBI analytical cross-sectional checklist. Each item was rated Yes, No, Unclear, or Not Applicable and supported by a source-based evidence note. Item responses were not summed into a numerical quality score.* Review-specific concern categories were used only to support interpretation and sensitivity analysis; lower concern required affirmative judgments across applicable critical domains; higher concern was assigned when at least one critical domain was rated No or at least two critical domains were Unclear; all other patterns were classified as some concerns.

Two reviewers independently completed item-level JBI appraisals for the revised evidence set against the corresponding full-text reports. Disagreements were resolved through discussion and consensus. Adjudicated judgments are reported in Supplementary Table S3a,b. Critical domains emphasized sampling frame and recruitment, autism ascertainment, measurement reliability, outcome ascertainment, follow-up, confounding where applicable, and analytical coherence.

### 2.7. Effect Measures and Observation Periods

For Axis A, the numerator was the number of unique people meeting the specified autism definition and the denominator was the corresponding eligible population with adequate ascertainment. Study-level exact-count proportions were accompanied by Wilson 95% confidence intervals (CIs). Administrative diagnoses, clinical diagnoses, and screening results retained their distinct interpretations. Studies counting assessments or admissions were not treated as person-level prevalence studies unless repeated observations could be resolved.

For Axis B proportions, the numerator is the number of unique autistic participants with at least one specified outcome during the stated observation period. It is not the total number of recurrent events. The denominator is the eligible autistic population to which that observation period applies, with losses and missing outcome data recorded. Recurrent-event rates require the appropriate person-time denominator and will remain separate from proportions. Censoring-adjusted cumulative risks and survival estimates retained their original metric rather than be converted into simple event fractions.

Lifetime histories, fixed retrospective periods, prospective follow-up, and unspecified periods were presented separately. Within these categories, duration and time origin also had to be comparable before synthesis. Risk ratios, odds ratios, hazard ratios, and incidence rate ratios were not pooled interchangeably. Adjusted and unadjusted results remained distinct. Selection among adjusted models considered the relevance of the confounders and potential adjustment for mediators or colliders, rather than simply choosing the largest covariate set. All selected adjustment sets and comparison groups are reported.

### 2.8. Statistical Analysis Plan

#### 2.8.1. Compatibility and Primary Model

Pooling required compatibility in recruitment, population, diagnostic definition and source, outcome, observation period, and effect measure. At least two independent cohort contributions are necessary but are not sufficient. No overall Axis A estimate or broad B1 proportion was calculated. Incompatible or single-study strata were presented through individual estimates and a structured narrative. Sparse compatible strata will emphasize the contributing studies and uncertainty rather than a single summary number. The currently selected Axis B evidence is presented as individual endpoint-specific estimates because a compatible set of independent contributions has not been established.

Compatible exact-count proportions were analyzed using a binomial generalized linear mixed model with a logit link and a normally distributed cohort-specific random intercept (Lin & Chu, 2020). Models were fitted by maximum likelihood using adaptive Gauss–Hermite quadrature with 15 integration points. The primary summary was the inverse logit of the fitted intercept, with a 95% profile-likelihood CI. This represents the median of the modeled distribution of cohort-specific proportions, rather than its marginal mean. The likelihood accommodated zero-event and all-event cohorts without a continuity correction. Optimization warnings, boundary estimates, and failure to obtain a valid profile interval were assessed; unstable models are not presented as reliable pooled results.

#### 2.8.2. Model Comparison and Heterogeneity

A sensitivity analysis used inverse-variance random-effects meta-analysis of logit-transformed proportions, with between-cohort variance estimated by restricted maximum likelihood (REML). Within-cohort sampling variance was defined as the sum of the reciprocals of the event and nonevent counts. Confidence intervals used modified Hartung–Knapp inference, with the residual variance multiplier constrained to be at least 1 and a t distribution with k − 1 degrees of freedom, where k is the number of independent contributions (Röver et al., 2015). Results were back-transformed to proportions. If a stratum contained boundary counts, this comparator was omitted rather than applying a continuity correction or dropping those cohorts.

Between-cohort variance was reported on the logit scale for each fitted model. For the inverse-variance comparator, Cochran’s Q used inverse within-cohort variance weights. The I^2 percentage was obtained by subtracting k − 1 from Q, dividing by Q, and multiplying by 100; negative values and the case of Q equal to zero were assigned zero. These comparator statistics were not transferred to the binomial model. Clinical and methodological differences were considered alongside these statistics. No prediction interval was calculated for these strata, which contained fewer than five independent contributions. This conservative reporting rule reflects the instability of prediction with few studies (Deeks et al., 2024). In particular, a two-contribution analysis received no prediction interval.

#### 2.8.3. Sensitivity Analyses and Missing Results

Sensitivity analyses examined alternative nonoverlapping cohort selections, source-supported numerator and denominator definitions, diagnostic restrictions, and relevant recruitment or measurement-bias domains. Leave-one-out analyses were used only when at least two contributions would remain; with two contributions, the individual estimates provided the influence comparison. Sensitivity findings were judged by changes in magnitude and precision. The current sparse strata did not support meaningful meta-regression, and incompatible settings were not combined merely to increase the number of observations for moderator analysis.

Risk of bias from missing results was examined by comparing reported methods, outcomes, associated publications, and protocols or registrations where available. The assessment considered unavailable reports, outcomes lacking usable estimates, and discrepancies in reported analyses. Formal funnel-plot asymmetry testing was not undertaken for the revised pooled strata because each contained only two cohorts, far below a defensible threshold for such testing.

#### 2.8.4. Certainty of Evidence

Certainty of evidence was assessed separately for each compatible population and outcome family using GRADE principles adapted to prevalence, prognosis, and observational association evidence (Iorio et al., 2015). Judgments considered risk of bias, inconsistency, indirectness, imprecision, and risk of missing results/publication bias. Because the revised meta-analysis deliberately avoids a global Axis A estimate and a broad pooled Axis B estimate, GRADE ratings were attached to the three compatible Axis A syntheses and to endpoint-specific Axis B evidence rather than to obsolete umbrella categories.

Two reviewers independently assessed certainty of evidence using the revised evidence structure, the adjudicated item-level JBI appraisals, and the prespecified GRADE domains. Domain judgments and downgrade decisions were resolved through discussion and consensus. No review-wide certainty rating was assigned.

#### 2.8.5. Reproducibility and Supplementary Materials

Reanalysis was implemented in R using lme4 for the binomial models (Bates et al., 2015) and metafor for inverse-variance synthesis (Viechtbauer, 2010). The completed package included the versioned analytical dataset, codebook, cohort map, extraction sources, amendment log, analysis scripts, and actual software versions and session information. Manuscript tables and figures were generated from the same frozen input, and study-level counts were reconciled with every displayed total. Participant totals were reported within compatible models; overlapping samples were not summed into an apparently unique review-wide population.

## 3. Results

### 3.1. Evidence Identification and Selection

The reconciled report register contained 300 candidate reports, of which 161 were retained and 139 were marked as excluded. The retained reports comprised 91 classified as providing quantitative evidence, 6 retained for separately interpreted estimates, 31 for narrative evidence, and 33 associated publications. These 161 reports mapped to 130 operational source-study units. Source-study units were not assumed to represent globally independent participant cohorts, because several publications used overlapping registers, services, or participant subsets (Table 1; Supplementary Table S1).

The reconciled report register and the working search ledger were maintained as distinct accounting levels. The working ledger included 13,883 records, 4371 duplicates, and 9512 records after deduplication. The report-level disposition register contained 300 candidate reports, of which 161 were retained and 139 excluded. Figure 1 and Supplementary Methods SM1 present these verified accounting levels without treating records, reports, source studies, cohorts, or analysis contributions as interchangeable units.

After source-level corrections and application of the revised compatibility rules, the numerical presentation comprised 37 contributions from 30 reports representing 29 source-study units: 24 Axis A entries and 13 Axis B entries. Six contributions, each from a separate cohort and report, entered three primary pooled analyses. The other 31 selected entries were presented individually; one was explicitly an assessment-record proportion. The remaining retained evidence and alternative contributions are traceable in the full selection ledger.

### 3.2. Study Characteristics

Axis A evidence represented substantially different recruitment processes: general prison populations, community youth justice referrals, offense-selected custodial populations, secure forensic services, intellectual-disability services, and forensic evaluation or referral populations. Administrative diagnostic codes, routine clinical diagnoses, structured assessments, historical pervasive developmental disorder (PDD) categories, and suspected autism were distinguished. The three pooled strata concerned clinically documented autism in specialist forensic intellectual-disability services, general forensic responsibility evaluations, and forensic youth referral services. These strata describe the composition of selected services or evaluation populations; they do not estimate a common prevalence across the criminal justice system.

The Axis B entries distinguished recruitment independently of justice involvement from recruitment after entry into forensic care. Police contact, charges, nonviolent convictions, violent convictions, intimate partner violence, cyber offending, and reconviction were retained as separate outcomes. Differences in observation windows, reported effect measures, adjustment sets, and overlapping Swedish population registers precluded a common Axis B summary. Participant denominators were not summed across the review or across overlapping outcomes.

### 3.3. Axis A: Autism in Justice and Forensic Populations

#### Correctional and Community Justice Populations

The three national cohorts in Bukten et al. (2024) recorded ICD-10 F84 diagnoses in 313 of 50,861 people imprisoned in Norway (0.62% (95% CI [0.55, 0.69])), 281 of 45,532 in Denmark (0.62% (95% CI [0.55, 0.69])), and 253 of 23,114 in Sweden (1.09% (95% CI [0.97, 1.24])). These were proportions with a recorded diagnosis during different calendar windows: 2010–2019, 2010–2018, and 2010–2013, respectively. They were therefore reported separately, without a common-period pooled prison estimate. They should not be interpreted as complete ascertainment of autism among all incarcerated people.

The later overlapping Norwegian cohort reported by Todd-Kvam et al. (2025) was not selected for an autism estimate because its supplementary diagnostic table assigned the label “Autism” to codes F80–F89 excluding F84. This conflicts with the intended autism construct. The verified Bukten et al. (2024) Norwegian cohort was used instead, and the two Norwegian samples were never counted as independent.

In the community out-of-court youth justice sample, 39 of 319 young people had confirmed autism (12.23% (95% CI [9.07, 16.28]); Murphy & Eley, 2026). Offense-selected custodial samples produced different descriptive estimates: 9.67% (95% CI [6.68, 13.79]) for 26 of 269 young men convicted of violent offenses (Billstedt et al., 2017), and 59.46% (95% CI [43.49, 73.65]) for 22 of 37 adolescents in a residential sexual-offense program (Sutton et al., 2013). These samples were not combined with general prison cohorts (Table 2).

### 3.4. Specialist Forensic Intellectual-Disability Services

Two clinical service cohorts contributed 61 autistic participants among 249 patients: 42/138 in Esan et al. (2015) and 19/111 in McKenna et al. (2025). The binomial random-effects estimate was 23.57% (95% CI [10.67, 42.69]), with between-cohort variance τ^2^ = 0.094 on the logit scale. Individual estimates were 30.43% and 17.12%, respectively. Both cohorts concerned specialist forensic intellectual-disability services and included mixed civil and criminal legal statuses; the estimand was service caseload composition. The broader cognitive-impairment sample of Stinson and Robbins (2014), which included traumatic brain injury and fetal alcohol syndrome and used historical PDD categories, was retained separately (35/235, 14.89% (95% CI [10.91, 20.01]).

### 3.5. Other Secure Forensic Services

No combined estimate was calculated for other secure forensic services. The administrative-code estimate in Sitter et al. (2025) was 18.50% (95% CI [16.85, 20.28]) (363/1962). The clinically recorded proportions in Eckert et al. (2017) were 7.69% (95% CI [3.57, 15.78]) in regular forensic care (6/78) and 18.33% (95% CI [10.56, 29.92]) in long-stay care (11/60 with diagnostic data). Haw et al. (2012) reported historical PDD in 29/231 patients (12.55% (95% CI [8.88, 17.45])). The 3/46 estimate in Dein et al. (2021), 6.52% (95% CI [2.24, 17.50]), represented existing Asperger diagnoses; incomplete participation in subsequent screening did not establish complete ASD ascertainment for the whole ward population. These differences were preserved rather than absorbed into an overall A2 estimate.Characteristics, sample sizes, and prevalence estimates for all secure forensic service cohorts are summarized in Table 3.

### 3.6. Forensic Evaluation and Youth Referral Populations

The two general forensic evaluation cohorts contributed 119 clinically confirmed cases among 15,235 individuals. Helverschou et al. (2015) contributed 45/3382 after removal of three historical diagnoses that the forensic experts replaced with other diagnoses; Boylu et al. (2025) contributed 74/11,853 using the corrected denominator. The pooled estimate was 0.89% (95% CI [0.36, 2.23]), with τ^2^ = 0.126. The individual proportions, 1.33% and 0.62%, differed despite broadly similar evaluation-based sampling. The Norwegian sample included participants aged 15–67 years, so this was not labelled an exclusively adult stratum. The Turkish article described the source as reports and individuals; explicit repeat-report deduplication was not documented and remains a denominator limitation.

Historical and differently selected evaluation samples were presented separately. Edberg et al. (2020) contributed 675/8442 under the study’s DSM-IV 299 definition (8.00% (95% CI [7.44, 8.59])). Kumagami and Matsuura (2009) explicitly separated general family courts, 11/335 (3.28% (95% CI [1.84, 5.78])), from a court department selected for particular offense presentations, 17/93 (18.28% (95% CI [11.74, 27.34]). The previous 28/428 aggregate was therefore not used for a general juvenile-court estimate. Rutten et al. (2022) identified 98 ASD records among 1799 pretrial assessment reports (5.45%); because person-level deduplication was not established, this fraction was kept at the assessment-record level and excluded from person-level pooling.

Two distinct London forensic youth referral services contributed 181 confirmed diagnoses among 770 young people: 75/327 in Loizidou et al. (2025) and 106/443 in Ross-Michaelides et al. (2024). The pooled estimate was 23.51% (95% CI [20.10, 27.08]). The fitted between-cohort variance was at the zero boundary (τ^2^ = 0), which does not establish absence of heterogeneity with only two cohorts. Suspected diagnoses and the associated publication from the same North West London cohort were not added as independent contributions. Further individual evaluation estimates, including competence-assessment and selected young-offender populations, are provided in Table 4.

### 3.7. Axis B: Justice Outcomes in Autism-Defined Populations

#### 3.7.1. Police Contact, Charges, and Nonviolent Outcomes

In the prospective Ontario cohort, 46 of 284 autistic adolescents and adults had at least one police interaction during 12–18 months of follow-up (16.20% (95% CI [12.37, 20.93]); Tint et al., 2017). This numerator counts people with an interaction, not the number of interactions, and the outcome does not imply offending in every case. In the South Carolina cohort, the extracted juvenile and adult charge histories identified 32 unique individuals among 606 participants (5.28% (95% CI [3.77, 7.36]); Y. Yu et al., 2021). The union was derived as 18 juvenile cases plus 20 adult cases minus 6 appearing in both systems. The variable observation period through ages 17–23 was not equated with the Ontario prospective window.

Historical PDD cohorts yielded different association measures: Mouridsen et al. (2008) reported a risk ratio of 0.50 (95% CI [0.34, 0.74]), whereas Mordre et al. (2011) reported an adjusted hazard ratio of 0.40 (95% CI [0.20, 0.90]) over approximately 30 years. These reported associations were not pooled or reconstructed from the raw fractions. In the Swedish youth study, adjusted hazard ratios for nonviolent convictions were 0.62 (95% CI [0.58, 0.67]) for males and 0.90 (95% CI [0.80, 1.01]) for females (Siponen et al., 2023). The sex-specific estimates came from one source study, not two independent studies.

For self-reported cyber offending during the preceding 12 months at age 17, Maras et al. (2024) reported an adjusted odds ratio of 0.94 (95% CI [0.42, 2.14]) in the selected model. The interval allowed associations in either direction. The weighted autism-subgroup percentage, 6.28% (reported 95% CI [2.48, 10.09]), was retained as a survey estimate and was not converted into an exact event count. Table 5a preserves the original metrics and observation periods.

#### 3.7.2. Violent Outcomes and Reconviction

Axis B describes eligible autism-defined cohorts identified through the review search and citation-searching procedures. Because the search was optimized for Axis A, and Axis B outcomes differed in definition and observation period, these findings are secondary and exploratory and should not be interpreted as an exhaustive estimate of justice-system involvement among autistic people.

Associations with violent outcomes varied with population, sex, outcome definition, and adjustment. Lundström et al. (2014) reported an adjusted odds ratio of 1.10 (95% CI [0.60, 1.90]). In Heeramun et al. (2017), the demographic- and family-adjusted risk ratio was 1.39 (95% CI [1.23, 1.58]); additional adjustment for ADHD and conduct disorder changed it to 0.85 (95% CI [0.75, 0.97]). Both models are shown because they address different adjustment conditions. Neither estimate was interpreted as a causal effect of autism.

Siponen et al. (2023) reported adjusted hazard ratios for violent convictions of 1.19 (95% CI [1.07, 1.32]) for males and 2.87 (95% CI [2.41, 3.42]) for females. For intimate partner violence resulting in arrest, R. Yu et al. (2019) reported a hazard ratio of 0.70 (95% CI [0.30, 1.60]) among men. These outcome-specific estimates were not combined with broader violence or conviction measures. The Swedish register studies were also not assumed to have disjoint participants.

Among individuals already sentenced to forensic psychiatric care, Edberg et al. (2022) reported a hazard ratio of 0.60 (95% CI [0.50, 0.80]) for reconviction associated with the recorded autism category. Follow-up began at the forensic psychiatric assessment and extended to first reconviction or censoring, for up to 17 years. This conditional forensic-cohort result does not estimate justice involvement among autistic people recruited independently of the justice system.

### 3.8. Sensitivity Analyses and Model Comparison

**Figure 2:**
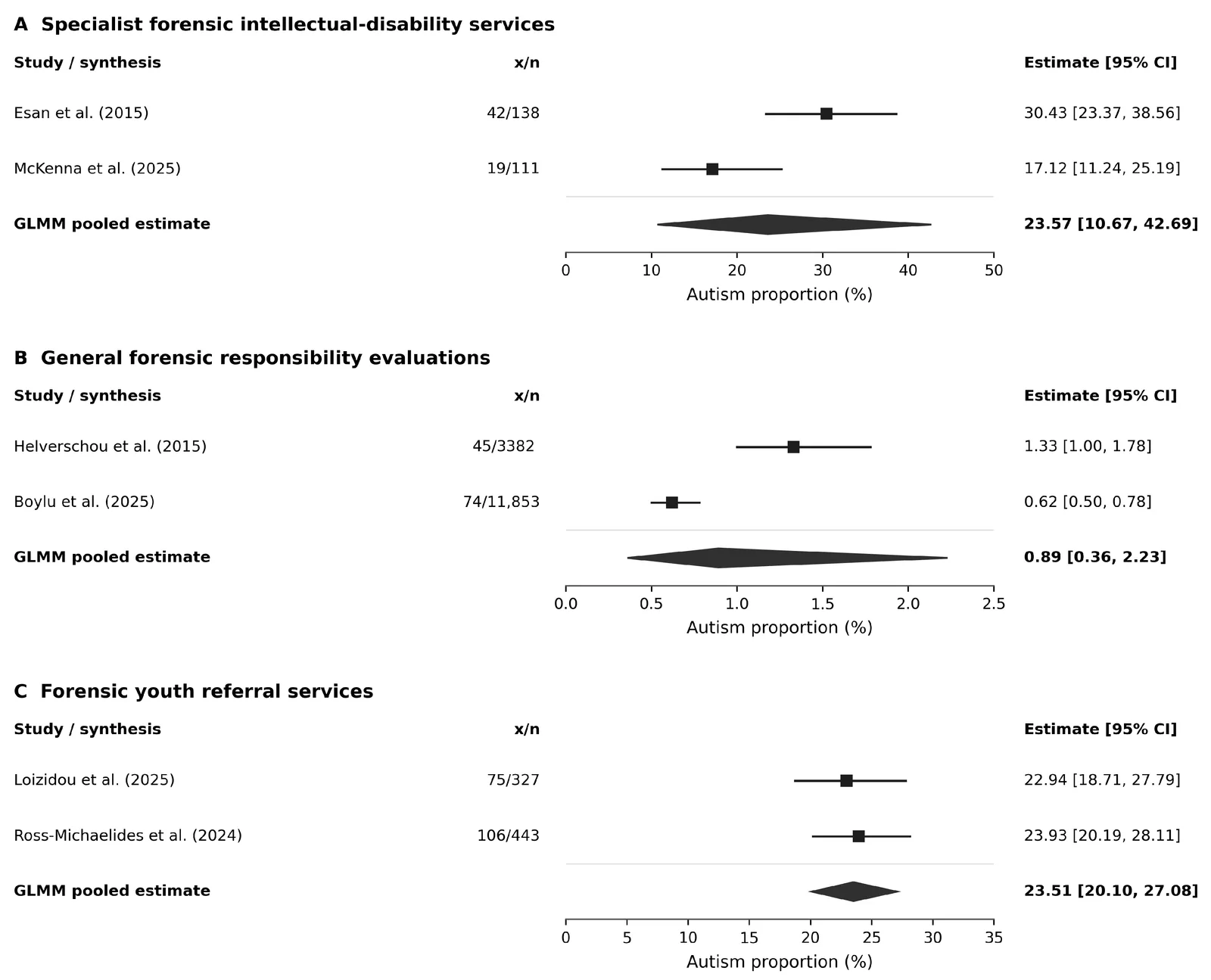
Clinical stratum estimates with individual and profile-likelihood intervals (Esan et al., 2015, McKenna et al., 2025; Helverschou et al., 2015; Boylu et al., 2025; Loizidou et al., 2025; Ross-Michaelides et al., 2024). Note: Squares show individual proportions with Wilson 95% CIs; diamonds show the binomial random-effects summaries with profile-likelihood 95% CIs. Horizontal scales differ by panel. The wider modified Hartung–Knapp intervals in Table 6 are necessary for interpreting uncertainty with only two cohorts per stratum. There is no overall pooled estimate.

The inverse-variance REML estimates were close to the GLMM point estimates, but modified Hartung–Knapp intervals were substantially wider. They were 23.46% (95% CI [0.26, 97.29]) for forensic intellectual-disability services, 0.91% (95% CI [0.01, 53.95]) for general forensic evaluations, and 23.51% (95% CI [9.45, 47.51]) for youth referral services. With two cohorts per model, the corresponding t distribution had one degree of freedom. The comparatively narrow profile-likelihood intervals therefore should not be interpreted as demonstrating stable precision across plausible inferential approaches (Table 6; Figure 2).

Comparator-model heterogeneity was substantial in the intellectual-disability and general evaluation strata: Q(1) = 5.77, *p* = 0.016, I^2^ = 82.7%, and Q(1) = 16.15, *p* < 0.001, I^2^ = 93.8%, respectively. For youth referral services, Q(1) = 0.10, *p* = 0.748, and I^2^ = 0%. These Q and I^2^ statistics belong to the inverse-variance comparator; they were not presented as GLMM statistics. All heterogeneity estimates were interpreted cautiously because each model contained only two cohorts.

Retaining all 48 historical Norwegian diagnoses instead of 45 currently confirmed diagnoses changed the general forensic evaluation estimate from 0.89% to 0.93% (95% CI [0.35, 2.49]). Removing either cohort from any primary model left a single study; individual estimates therefore served as the influence assessment, without fitting a one-study random-effects model. Under a strict requirement for explicitly documented person-level deduplication, the Turkish report would not support that pooled stratum, leaving the Norwegian estimate alone.

Other source-supported sensitivities were retained at the individual-study level. The alternative, probably overlapping Walters et al. (2013) sample gave 27/43 rather than Sutton’s 22/37 and was never added to it. Inclusion of suspected autism in Murphy and Eley (2026) gave 123/319, a different diagnostic construct from the 39 confirmed cases. For the one missing long-stay diagnostic record in Eckert et al. (2017), the complete-sample scenarios were 11/61 and 12/61. In the cyber-offending study, earlier adjustment models gave odds ratios of 1.13 (95% CI [0.53, 2.41]) and 1.04 (95% CI [0.49, 2.22]), compared with 0.94 in the selected model (Supplementary Table S5).

All three GLMMs converged. Re-estimation using 61 rather than 15 quadrature points and independent adaptive integration reproduced the pooled estimates and confidence limits to well beyond the displayed precision; the largest checked log-likelihood difference was below 1.3 × 10^−10^. No prediction intervals, meta-regressions, subgroup interaction tests, or funnel-asymmetry tests were calculated for these two-cohort strata.

### 3.9. Risk of Bias and Certainty of Evidence

Two reviewers independently completed the revised JBI appraisal, which covered 22 Axis A study contributions and 10 Axis B studies represented in the current item-level assessment set; disagreements were resolved by consensus. For Axis A, 5 contributions were classified as lower concern, 12 as some concerns, and 5 as higher concern. The most consequential limitations were nonrepresentative or highly selected sampling frames, incomplete description of diagnostic procedures, use of routine administrative diagnoses without active case finding, and uncertainty in coverage of the intended denominator.

For Axis B, seven studies were classified as lower concern, two as some concerns, and one as higher concern, after reviewer consensus. The principal concerns involved attrition or baseline outcome status in prospective survey evidence, residual confounding, self-reported or incompletely described outcomes, and heterogeneity in diagnostic and justice-outcome definitions. Detailed item-level judgments are provided in Supplementary Table S3a,b.

GRADE certainty was very low for the pooled estimate from specialist forensic intellectual-disability services (23.57%, 95% CI [10.67, 42.69]) because the evidence comprised only two service cohorts and was limited by risk of bias, inconsistency, and imprecision. Certainty was also very low for general forensic responsibility evaluations (0.89%, 95% CI [0.36, 2.23]) because two cohorts differed substantially and the estimate remained sensitive to source-level and deduplication decisions.

The forensic youth referral synthesis (23.51%, 95% CI [20.10, 27.08]) was rated low certainty. The two cohorts were internally coherent and statistically consistent, but both came from closely related specialist London referral pathways, limiting transferability, and the evidence base remained small. Axis B endpoint families were rated very low certainty overall. Police interaction was represented by a single prospective cohort with attrition and a broad contact definition; charge/conviction and violent-outcome evidence used non-combinable metrics across different populations, eras, and adjustment sets; cyber offending was supported by a single imprecise estimate; and recidivism was represented by one forensic cohort. Domain-level rationales are reported in Supplementary Table S7.

## 4. Discussion

### 4.1. Principal Findings

This review does not support a single pooled estimate for autism across the criminal-justice system. The defensible quantitative result is instead a set of setting-specific estimates. Autism accounted for approximately one quarter of caseloads in two specialist forensic populations: 23.57% in forensic intellectual-disability services and 23.51% in forensic youth referral services. By contrast, clinically confirmed autism in general forensic responsibility evaluations was estimated at 0.89%. Administrative prison-register estimates were similarly low, between 0.62% and 1.09%, but they measure recorded diagnoses rather than complete diagnostic ascertainment. The magnitude of the estimate therefore changes with the pathway into the sample and with how autism is identified.

This pattern clarifies why the very broad ranges reported in previous systematic reviews should not be read as estimates of one underlying prevalence. Chester et al. (2025) reported autism proportions from 0% to 60% across justice and forensic settings, while earlier reviews similarly emphasized methodological and setting heterogeneity. Our analysis advances that literature by treating heterogeneity as a design feature rather than merely a statistic; incompatible recruitment pathways and diagnostic constructs were not pooled simply because they all occurred somewhere within a justice or forensic system.

The three pooled analyses were intentionally sparse. Each contained only two cohorts. Their GLMM point estimates were stable to numerical re-estimation, but the modified Hartung–Knapp sensitivity intervals were much wider, especially for forensic intellectual-disability services and general forensic evaluations. Thus, the apparent precision of profile-likelihood intervals should not be mistaken for a large evidence base. The principal inference is about the location and context of the observed estimates, not about universal prevalence. The certainty assessment reinforces this caution: specialist forensic intellectual-disability services and general forensic responsibility evaluations were rated very low certainty, whereas the forensic youth referral synthesis was rated low certainty because of indirectness and the small, geographically concentrated evidence base.

### 4.2. Why Setting and Ascertainment Matter

High proportions in specialist services are compatible with referral and service-selection mechanisms. Forensic intellectual-disability services are designed for people with complex neurodevelopmental, psychiatric, behavioral, and legal needs; forensic child and adolescent services likewise receive selected referrals rather than population-representative youth. Their caseload composition should therefore not be extrapolated to prisons, courts, or autistic people in the community.

Conversely, low administrative prison estimates may underestimate underlying autism when diagnoses are absent from registers or were never established. A recorded ICD code answers a different question from systematic case finding. The present review therefore avoids interpreting the Scandinavian prison estimates as evidence that autism is rare among incarcerated people. Equally, offense-selected residential or custodial samples, including the high estimate in the Sutton et al. (2013) sample, cannot be generalized to ordinary prison populations.

Historical diagnostic categories add another layer. Asperger syndrome, autistic disorder, broad DSM-IV 299 categories, and PDD are not interchangeable with contemporary autism spectrum disorder. Retaining original definitions reduced the temptation to manufacture comparability across decades. This choice narrowed the number of poolable studies but improved interpretability.

### 4.3. Justice Outcomes in Autism-Defined Populations

Axis B did not yield a coherent single direction of association. Prospective police interaction, charge histories, nonviolent convictions, violent convictions, intimate-partner-violence arrest, cyber offending, and reconviction after forensic care differed in population and meaning. Some reported relative estimates were below 1, some were close to the null, and some violence estimates were above 1. These findings do not justify describing autism itself as uniformly increasing or decreasing criminal behavior.

Adjustment choices were particularly consequential. In Heeramun et al. (2017), the association with violent conviction changed from a risk ratio above 1 after demographic and family adjustment to below 1 after additional adjustment for ADHD and conduct disorder. Those models answer different conditional questions, and the latter covariates may lie on complex causal pathways. The change illustrates why the largest covariate set is not automatically the most causally informative model.

Sex-specific findings also require restraint. Siponen et al. (2023) reported different hazard ratios for males and females, but these estimates came from the same national source study and are not independent replications. Similarly, Swedish register studies may share underlying populations. Treating every published estimate as an independent study would artificially inflate the evidence base.

### 4.4. Clinical and Criminal-Justice Implications

The findings favor targeted identification and accommodation rather than assumptions based on a universal prevalence figure. Services with concentrated neurodevelopmental needs may require reliable diagnostic pathways, communication adjustments, sensory accommodations, staff training, and coordination across intellectual-disability, autism, mental-health, and legal services. In general prisons and court pathways, low proportions of recorded diagnoses should not be taken as evidence that systematic recognition is unnecessary.

The results also argue against stigmatizing inferences from forensic samples to autistic people generally. Forensic cohorts are selected precisely because participants have entered specialized legal or clinical pathways. Evidence from autism-defined population cohorts is more appropriate for questions about subsequent justice involvement, and even there outcomes and adjustment strategies vary. Clinical assessment should remain individualized and should consider co-occurring conditions, developmental history, social context, victimization, and vulnerability, rather than treating autism as a proxy for offending risk.

### 4.5. Strengths and Limitations

A major strength is the explicit separation of records, reports, source studies, cohorts, and effect contributions. Potentially overlapping publications were mapped conservatively, and alternative reports such as Sutton et al. (2013) and Walters et al. (2013) were not counted as independent. Exact counts, weighted survey estimates, record-level denominators, historical diagnoses, and model-based associations were also kept distinct. These decisions reduce pseudo-replication and improve the clinical meaning of pooled estimates.

The review nevertheless has important limitations. First, the compatible meta-analytic strata were extremely small, with only two cohorts each. Heterogeneity estimates and random-effects uncertainty are therefore unstable, prediction intervals were not appropriate, and meta-regression would have been uninformative. Second, several included estimates depended on routine clinical documentation, administrative coding, historical diagnostic categories, or highly selected forensic pathways rather than standardized findings from active cases. These differences limit direct comparability and transferability across settings.

Third, the evidence base spans jurisdictions, periods, diagnostic systems, and legal pathways that differ substantially in referral thresholds and service organization. Even apparently similar labels such as forensic evaluation or secure care may therefore represent different target populations. Fourth, each pooled synthesis contained only two cohorts, and several Axis B outcomes were represented by single studies or non-combinable effect measures. The resulting certainty ratings appropriately reflect the limited amount, precision, consistency, and transferability of the available evidence.

### 4.6. Research Priorities

Future studies should report the sampling frame, recruitment coverage, diagnostic source, unique-person denominator, observation period, legal status, and co-occurring intellectual disability in sufficient detail to support synthesis. Prospective autism-defined cohorts linked to official justice records can strengthen temporal interpretation, while justice-system studies need systematic case-finding designs if the aim is true prevalence rather than recorded diagnosis.

For meta-analysis, the next useful advance is not a larger indiscriminate pool. It is accumulation of independent cohorts within comparable settings using comparable diagnostic definitions and endpoints. Shared registers and repeated service publications should carry persistent cohort identifiers. Where sex, intellectual disability, or co-occurring psychiatric conditions are examined, sufficiently large independent datasets are needed before moderator analyses can be interpreted reliably.

## 5. Conclusions

Autism in criminal-justice and forensic populations cannot be represented credibly by one prevalence estimate. In this review, pooled proportions ranged from 0.89% in general forensic responsibility evaluations to approximately 23.5% in specialist forensic intellectual-disability and youth referral services, while administrative prison estimates were below 1.1%. These differences reflect distinct recruitment pathways and ascertainment methods as well as underlying populations. Justice outcomes in the autism-defined cohorts were similarly endpoint-specific and the present findings do not support a unitary association between autism and offending. Future synthesis of evidenceshould prioritize population definition, diagnostic validity, cohort independence, and outcome comparability over broad pooling. With regard to practice, the present findings support context-sensitive identification and accommodation while cautioning against extrapolating from selected forensic samples to autistic people generally.

## Figures and Tables

**Table 1:** Report, Study, and Analysis Counts.

**Unit**	**Count**	**Interpretation**
Registered candidate reports	300	Report-level disposition register
Retained reports	161	91 quantitative reports; 6 reports providing separate estimates; 31 narrative reports; 33 associated reports
Excluded report records	139	A specific primary reason for exclusion requires documentation for 94 records
Operational source-study units	130	Participant independence is not assumed across units
Selected numerical reports	30	Represent 29 source-study units
Selected contributions	37	24 contributions to Axis A; 13 contributions to Axis B
Pooled clinical analyses	3	Each analysis includes 2 cohorts
Contributions to pooled analyses	6	Representing 6 separate cohorts and reports
Selected individual entries	31	Including one assessment-record fraction
Working search records	13,883	The original mapping from search results to screening records is incomplete
Working duplicates	4371	Based on the author-validated working ledger
Working records after deduplication	9512	Do not constitute a fully reconstructed PRISMA flow

*Note.* Reports, source-study units, contributions, and analytical entries represent distinct counting units and should not be treated as interchangeable. Counts are not additive across rows. PRISMA = Preferred Reporting Items for Systematic Reviews and Meta-Analyses.

**Table 2:** Reported Autism Frequencies Across Selected Justice Settings.

Source	Recruitment and Diagnosis	x/*n*	% [95% CI]
Bukten et al. (2024), Norway	General prisons; administrative F84; 2010–2019	313/50,861	0.62 [0.55, 0.69]
Bukten et al. (2024), Denmark	General prisons; administrative F84; 2010–2018	281/45,532	0.62 [0.55, 0.69]
Bukten et al. (2024), Sweden	General prisons; administrative F84; 2010–2013	253/23,114	1.09 [0.97, 1.24]
Murphy and Eley (2026)	Community youth justice; confirmed ASC	39/319	12.23 [9.07, 16.28]
Billstedt et al. (2017)	Violent-offense-selected young men	26/269	9.67 [6.68, 13.79]
Sutton et al. (2013)	Sexual-offense-selected adolescents	22/37	59.46 [43.49, 73.65]

*Note.* CI = confidence interval; ASC = autism spectrum condition. Values and confidence intervals are reproduced as supplied.

**Table 3:** Secure Forensic Service Samples.

Source	Recruitment and Diagnosis	x/*n*	% [95% CI]
Esan et al. (2015)	Forensic ID service; clinical ASD	42/138	30.43 [23.37, 38.56]
McKenna et al. (2025)	Forensic ID service; clinical ASD	19/111	17.12 [11.24, 25.19]
Stinson and Robbins (2014)	Broad cognitive impairment; historical PDD	35/235	14.89 [10.91, 20.01]
Sitter et al. (2025)	Secure services; administrative ASD	363/1962	18.50 [16.85, 20.28]
Eckert et al. (2017), regular care	Regular secure care; clinical ASD	6/78	7.69 [3.57, 15.78]
Eckert et al. (2017), long-stay care	Long-stay care; clinical ASD	11/60	18.33 [10.56, 29.92]
Haw et al. (2012)	Secure hospital; historical PDD	29/231	12.55 [8.88, 17.45]
Dein et al. (2021)	Medium-secure wards; known Asperger diagnoses	3/46	6.52 [2.24, 17.50]

*Note.* CI = confidence interval; ID = intellectual disability; ASD = autism spectrum disorder; PDD = pervasive developmental disorder.

**Table 4:** Forensic Evaluation and Referral Samples.

Source	Recruitment and Diagnosis	x/*n*	% [95% CI]
Helverschou et al. (2015)	General forensic evaluations; 45 confirmed diagnoses	45/3382	1.33 [1.00, 1.78]
Boylu et al. (2025)	General forensic responsibility evaluations	74/11,853	0.62 [0.50, 0.78]
Edberg et al. (2020)	Standardized forensic assessments; DSM-IV 299	675/8442	8.00 [7.44, 8.59]
Kumagami and Matsuura (2009), general courts	General family courts; historical PDD	11/335	3.28 [1.84, 5.78]
Kumagami and Matsuura (2009), specialist court	Offense-selected court; historical PDD	17/93	18.28 [11.74, 27.34]
Rutten et al. (2022)	Juvenile pretrial assessment records; ASD	98/1799	5.45 [not calculated]
Bath et al. (2015)	Youth competence assessment; historical PDD	19/323	5.88 [3.80, 9.00]
Siponmaa et al. (2001)	Selected young-offender evaluations; historical PDD	19/126	15.08 [9.87, 22.35]
Loizidou et al. (2025)	Tavistock/Portman youth referrals; confirmed ASD	75/327	22.94 [18.71, 27.79]
Ross-Michaelides et al. (2024)	North West London youth referrals; confirmed ASC	106/443	23.93 [20.19, 28.11]

*Note.* CI = confidence interval; ASD = autism spectrum disorder; ASC = autism spectrum condition; PDD = pervasive developmental disorder; DSM-IV = *Diagnostic and Statistical Manual of Mental Disorders*, fourth edition.

**Table 5:** (**a**) Police, Charge, and Nonviolent Axis B Estimates. (**b**) Violent Outcomes and Reconviction Axis B Estimates.

**(a)**			
**Source**	**Outcome and Window**	**Metric**	**Estimate [95% CI]**
Tint et al. (2017)	Police interaction; prospective 12–18 months	%	16.20 [12.37, 20.93] 46/284
Y. Yu et al. (2021)	Any juvenile/adult charge history; through ages 17–23	%	5.28 [3.77, 7.36] 32/606
Mouridsen et al. (2008)	Criminal conviction; historical PDD cohort	RR	0.50 [0.34, 0.74]
Mordre et al. (2011)	Delinquency/conviction; approximately 30-year follow-up	HR	0.40 [0.20, 0.90]
Siponen et al. (2023)	Nonviolent conviction; males, ages 15–20	HR	0.62 [0.58, 0.67]
Siponen et al. (2023)	Nonviolent conviction; females, ages 15–20	HR	0.90 [0.80, 1.01]
Maras et al. (2024)	Cyber offending; preceding 12 months at age 17	OR	0.94 [0.42, 2.14]
(**b**)			
**Source**	**Outcome and Window**	**Metric**	**Estimate [95% CI]**
Lundström et al. (2014)	Violent criminality; registry outcome through 2009	OR	1.10 [0.60, 1.90]
Heeramun et al. (2017)	Violent conviction; ages 15–27, through 2011	RR	1.39 [1.23, 1.58]
Siponen et al. (2023)	Violent conviction; males, ages 15–20	HR	1.19 [1.07, 1.32]
Siponen et al. (2023)	Violent conviction; females, ages 15–20	HR	2.87 [2.41, 3.42]
R. Yu et al. (2019)	Intimate partner violence arrest; men, mean follow-up 4.8 years	HR	0.70 [0.30, 1.60]
Edberg et al. (2022)	Reconviction after forensic assessment; up to 17 years	HR	0.60 [0.50, 0.80]

*Note.* CI = confidence interval; RR = risk ratio; HR = hazard ratio; OR = odds ratio; PDD = pervasive developmental disorder. For percentage estimates, x/*n* is shown below the estimate.

**Table 6:** Primary Clinical Syntheses and Small-Sample Model Comparison.

Population	x/*n*	GLMM % [95% CI]	τ^2^	REML–mHK % [95% CI]	I^2^%
Specialist forensic intellectual-disability services	61/249	23.57 [10.67, 42.69]	0.094	23.46 [0.26, 97.29]	82.7
General forensic responsibility evaluations	119/15,235	0.89 [0.36, 2.23]	0.126	0.91 [0.01, 53.95]	93.8
Forensic youth referral services	181/770	23.51 [20.10, 27.08]	0.000	23.51 [9.45, 47.51]	0.0

*Note.* CI = confidence interval; GLMM = generalized linear mixed model; REML = restricted maximum likelihood; mHK = modified Hartung–Knapp adjustment.

## Data Availability

The deidentified effect-level dataset, decision log, and analysis code are available from the corresponding author upon reasonable request.

## Supplementary Materials

The following supporting information can be downloaded at https://www.mdpi.com/article/10.3390/bs16101923/s1, PRISMA 2020 Checklist; Document S1: Supplementary Methods SM1 and SM2; Table S1: Reconciled report and analysis accounting; Table S2: Quantitative evidence dataset; Table S3a,b: Adjudicated JBI critical appraisal judgments; Table S4: Primary and comparator meta-analytic models; Table S5: Sensitivity analyses; Table S6: Missing results assessment; Table S7: GRADE evidence profile; Figure S1: JBI risk-of-bias traffic light plot; Note S1: Interpretive guide.
