# Supplementary material for: Autism in Criminal-Justice and Forensic Settings: A Systematic Review and Meta-Analysis with an Exploratory Bidirectional Synthesis
Source: Behav Sci (Basel). 2026 Oct 16;16(10):1923. doi: 10.3390/bs16101923 (PMC13701589; doi:10.3390/bs16101923)
Supplement: Supplementary file 1 [file behavsci-16-01923-s001.zip › behavsci-4589971-supplementary.pdf]

**Supplementary Materials**

**Autism in Criminal Justice and Forensic Populations: Revised Evidence Synthesis**

These supplementary materials correspond to the revised synthesis framework reported in the main manuscript. They do not contain the superseded overall Axis A prevalence meta-analysis, broad Axis B pooling, meta-regression, prediction intervals, or funnel-asymmetry analyses from earlier analytic versions.

## Supplementary Methods SM1. Search Sources and Record Accounting

The review used PubMed/MEDLINE, Lens.org, NCJRS, Scopus, Web of Science Core Collection, and ProQuest Central Premium, supplemented by citation searching. PsycINFO was inaccessible; Embase and Criminal Justice Abstracts were not searched. The preserved audit materials document the source-level counts below and the concept blocks used (autism terms combined with criminal-justice, forensic, correctional, court, offending, violence, arrest, conviction, and related terms). However, the exact verbatim historical database-specific strings, field tags, and all interface settings were not preserved for every source and cannot be reconstructed reliably after the fact. We therefore report only verifiable search details and do not retrospectively invent exact syntax. The revised searches were completed through September 2026. This limitation is now explicit because it constrains full PRISMA-S reproducibility.

| Source | Date | Documented records / notes |
|---|---|---|
| PubMed/MEDLINE | 25 Aug 2026 | 370 records |
| Lens.org | 25 Aug 2026 | 1,216 records |
| NCJRS | 26 Aug 2026 | 32 recovered records; original gross total not preserved |
| Scopus | 22 Sep 2026 | 7,407 exported |
| Web of Science Core | 22 Sep 2026 | 1,753 displayed; 1,754 exported because one row was repeated at a batch boundary |
| ProQuest Central Premium | 22 Sep 2026 | 3,499 displayed after Scholarly Journals filter; 3,500 exported; extra export row requires archival verification |
| Citation searching | Aug-Sep 2026 | Used to identify linked and additional reports |

Working search ledger: 13,883 records before deduplication; 4,371 duplicate records removed; 9,512 records remained after deduplication. This is a preserved historical working-ledger total and is not the arithmetic sum of the revision-stage source counts in the table above. The September source audit documents the expanded/revised searches, whereas the original database-level crosswalk underlying the 13,883-record ledger was not preserved. The preserved search and screening logs also do not permit exact reconstruction of the historical title/abstract exclusion count or retrieval-failure count. No missing database-level or stage count was derived by subtraction.

Search-strategy reproducibility note. Available archival information supports the source names, search dates, documented export/display counts, the ProQuest Scholarly Journals filter, and the concept blocks reported above. Exact historical query syntax and field restrictions are unavailable for some interfaces. Consequently, this supplement distinguishes verifiable archival details from information that cannot be recovered, rather than presenting reconstructed strings as if they were contemporaneous search records.

## Supplementary Methods SM2. Revision-Stage Analytical Amendments

The review was not prospectively registered. After examination of the evidence and additional September 2026 searches, the synthesis framework was revised on 28-29 September 2026. The revisions narrowed population and diagnostic strata, made cohort-overlap rules explicit, removed the overall Axis A and broad B1 pooled estimates, corrected source-level discrepancies, and replaced the earlier broad random-effects approach with population-compatible binomial GLMMs. These decisions are revision-stage amendments and are not presented as prospectively specified analyses.

Pooling required substantive compatibility in recruitment frame, represented population, autism ascertainment, outcome definition, observation period, and unit of analysis. A minimum of two studies was necessary but not sufficient for pooling. Axis B outcomes were retained as endpoint-specific individual estimates when metrics or target populations were not sufficiently compatible.

## Supplementary Table S1. Reconciled Report and Analysis Accounting

| Unit | n | Interpretation |
|---|---|---|
| Candidate reports in reconciled disposition register | 300 | Report-level register |
| Retained reports | 161 | 91 quantitative; 6 separately interpreted; 31 narrative; 33 associated reports |
| Excluded report records | 139 | Primary reason recoverable for 45 reports; 94 historical exclusions lack sufficient documentation for reliable reconstruction |
| Operational source-study units represented by retained reports | 130 | Not globally independent |
| Reports contributing to current numerical presentation | 30 | Current revised evidence set |
| Source-study units contributing to current numerical presentation | 29 | Accounts for linked/overlapping reports |
| Selected analysis contributions | 37 | 24 Axis A and 13 Axis B contributions |
| Contributions entering pooled analyses | 6 | Three population-compatible pools, each k = 2 |

| Unit | n | Interpretation |
|---|---|---|
| Individually presented contributions | 31 | Not combined because of population, outcome, metric, diagnostic, or dependence differences |

Note. Records, reports, source studies, cohorts, and analysis contributions are distinct units. Counts therefore should not be added across levels.

### Supplementary Table S2. Quantitative Evidence Used in the Revised Synthesis

| Study / cohort | Axis | Population / outcome | Events/N | Estimate | Use |
|---|---|---|---|---|---|
| Bukten et al., 2024 | A1 | General prison registry | 313/50,861 | 0.62% [0.55, 0.69] | Individual |
| Denmark registry cohort | A1 | General prison registry | 281/45,532 | 0.62% | Individual |
| Sweden registry cohort | A1 | General prison registry | 253/23,114 | 1.09% [0.97, 1.24] | Individual |
| Murphy & Eley, 2026 | A1 | Community youth justice | 39/319 | 12.23% | Individual |
| Billstedt et al., 2017 | A1 | Offense-selected custody | 26/269 | 9.67% | Individual |
| Sutton et al., 2013 | A1 | Offense-selected residential | 22/37 | 59.46% | Individual |
| Esan et al., 2015 | A2 | Forensic ID service | 42/138 | 30.43% | Pool 1 |
| McKenna et al., 2025 | A2 | Forensic ID service | 19/111 | 17.12% | Pool 1 |
| Stinson & Robbins, 2014 | A2 | Cognitive-impairment forensic service | 35/235 | 14.89% | Individual |
| Sitter et al., 2025 | A2 | National forensic care | - | 18.50% | Individual |
| Eckert et al., 2017 | A2 | Regular secure service | - | 7.69% | Individual |
| Eckert et al., 2017 | A2 | Long-stay secure service | 11/60 | 18.33% | Individual |
| Haw et al., 2012 | A2 | Secure service; historical PDD | - | 12.55% | Individual |

| Study / cohort | Axis | Population / outcome | Events/N | Estimate | Use |
|---|---|---|---|---|---|
| Dein et al., 2021 | A2 | Secure service; Asperger diagnosis | - | 6.52% | Individual |
| Helverschou et al., 2015 | A3 | General forensic responsibility evaluations | 45/3,382 | 1.33% | Pool 2 |
| Boylu et al., 2025 | A3 | General forensic responsibility evaluations | 74/11,853 | 0.62% | Pool 2 |
| Edberg et al., 2020 | A3 | Standardized forensic assessments | 675/8,442 | 8.00% | Individual |
| Kumagami & Matsuura, 2009 | A3 | General court sample | 11/335 | 3.28% | Individual |
| Kumagami & Matsuura, 2009 | A3 | Specialist offense-selected court | 17/93 | 18.28% | Individual |
| Rutten et al., 2022 | A3 | Assessment records | 98/1,799 | 5.45% | Assessment-record fraction only |
| Loizidou et al., 2025 | A3 | Forensic youth referrals | 75/327 | 22.94% | Pool 3 |
| Ross-Michaelides et al., 2024 | A3 | Forensic youth referrals | 106/443 | 23.93% | Pool 3 |
| Tint et al., 2017 | B | Police interaction | 46/284 | 16.20% [12.37, 20.93] | Individual |
| Yu et al., 2021 | B | Charge history | 32/606 | 5.28% | Individual |
| Mouridsen et al., 2008 | B | Criminal outcome | - | RR 0.50 [0.34, 0.74] | Individual |
| Mordre et al., 2011 | B | Criminal outcome | - | HR 0.40 [0.20, 0.90] | Individual |
| Siponen et al., 2023 | B | Nonviolent criminality | - | HR male 0.62 [0.58, 0.67]; female 0.90 [0.80, 1.01] | Individual |

| Study / cohort | Axis | Population / outcome | Events/N | Estimate | Use |
|---|---|---|---|---|---|
| Maras et al., 2024 | B | Cyber offending | - | OR 0.94 [0.42, 2.14] | Individual |
| Lundström et al., 2014 | B | Violent conviction/criminality | - | OR 1.10 [0.60, 1.90] | Individual |
| Heeramun et al., 2017 | B | Violent conviction/criminality | - | RR 1.39 [1.23, 1.58]; 0.85 [0.75, 0.97] after ADHD/CD adjustment | Individual |
| Siponen et al., 2023 | B | Violent criminality | - | HR male 1.19 [1.07, 1.32]; female 2.87 [2.41, 3.42] | Individual |
| Yu et al., 2019 | B | IPV arrest | - | HR 0.70 [0.30, 1.60] | Individual |
| Edberg et al., 2022 | B | Reconviction after forensic assessment | - | HR 0.60 [0.50, 0.80] | Individual |
| Bath et al., 2015 | A3 | Youth competence assessment; historical PDD | 19/323 | 5.88% [3.80, 9.00] | Individual |
| Siponmaa et al., 2001 | A3 | Selected young-offender evaluations; historical PDD | 19/126 | 15.08% [9.87, 22.35] | Individual |

Note. This table summarizes the quantitative contributions explicitly reported in the revised manuscript. Some report-level contributions are represented in the accounting register but are not pooled because they are historical diagnostic constructs, alternative overlapping samples, sensitivity variants, or otherwise analytically non-combinable.

## Supplementary Tables S3a-S3b. JBI Consensus Judgments

Two reviewers independently appraised each contributing study component using the design-appropriate JBI checklist. Disagreements were resolved through discussion and consensus. Items were not summed into a numerical quality score. Review-level concern categories were used only as interpretive and sensitivity flags.

| Study | Q1 | Q2 | Q3 | Q4 | Q5 | Q6 | Q7 | Q8 | Q9 | Consensus |
|---|---|---|---|---|---|---|---|---|---|---|
| Bukten 2024 | Y | Y | Y | Y | Y | Y | Y | Y | Y | Lower concern |
| Murphy & Eley 2026 | Y | Y | Y | Y | Y | Y | U | Y | Y | Some concerns |
| Billstedt 2017 | Y | Y | Y | Y | Y | Y | Y | Y | Y | Lower concern |
| Sutton 2013 | N | N | N | Y | N | Y | Y | Y | Y | Higher concern |

| Study | Q1 | Q2 | Q3 | Q4 | Q5 | Q6 | Q7 | Q8 | Q9 | Consensus |
|---|---|---|---|---|---|---|---|---|---|---|
| Esan 2015 | Y | Y | N | Y | Y | Y | Y | Y | Y | Some concerns |
| McKenna 2025 | Y | Y | N | Y | Y | Y | Y | U | Y | Some concerns |
| Stinson & Robbins 2014 | Y | Y | Y | Y | Y | U | U | Y | Y | Higher concern |
| Sitter 2025 | Y | Y | Y | Y | Y | Y | Y | Y | Y | Lower concern |
| Eckert 2017 regular | Y | Y | N | Y | Y | Y | Y | Y | Y | Some concerns |
| Eckert 2017 long-stay | Y | Y | N | Y | Y | Y | Y | Y | U | Some concerns |
| Haw 2012 | Y | Y | Y | Y | Y | U | Y | Y | Y | Some concerns |
| Dein 2021 | Y | Y | Y | Y | Y | N | U | Y | Y | Higher concern |
| Helverschou 2015 | Y | Y | Y | Y | Y | Y | Y | Y | Y | Lower concern |
| Boylu 2025 | Y | Y | Y | Y | Y | Y | U | Y | Y | Some concerns |
| Edberg 2020 | Y | Y | Y | Y | Y | Y | Y | Y | Y | Lower concern |
| Kumagami general 2009 | N | N | Y | Y | N | Y | U | Y | U | Higher concern |
| Kumagami specialist 2009 | N | N | N | Y | N | Y | U | Y | U | Higher concern |
| Rutten 2022 | Y | Y | Y | Y | Y | Y | Y | Y | Y | Lower concern |
| Bath 2015 | Y | Y | Y | Y | Y | U | Y | Y | Y | Some concerns |
| Siponmaa 2001 | Y | Y | N | Y | Y | U | Y | Y | Y | Some concerns |
| Loizidou 2025 | Y | Y | Y | Y | Y | Y | Y | Y | Y | Lower concern |

| Study | Q1 | Q2 | Q3 | Q4 | Q5 | Q6 | Q7 | Q8 | Q9 | Consensus |
|---|---|---|---|---|---|---|---|---|---|---|
| Ross-Michaelides 2024 | Y | Y | Y | Y | Y | Y | Y | U | Y | Some concerns |

Table S3a note. JBI prevalence checklist: Q1 appropriate sample frame; Q2 appropriate sampling; Q3 adequate sample size; Q4 participants/setting described; Q5 sufficient coverage; Q6 valid autism identification; Q7 reliable/consistent measurement; Q8 appropriate analysis; Q9 adequate response/management. Consensus categories follow the stated rule: all applicable critical domains affirmative = lower concern; at least one critical domain No or at least two critical domains Unclear = higher concern; otherwise = some concerns. Bukten 2024 and Sitter 2025 are therefore classified as lower concern because all displayed items are affirmative. Material evidence notes: routine administrative diagnosis can still limit ascertainment/transferability and is handled in GRADE even when the JBI item pattern is affirmative; Boylu 2025 retains some concerns because person-level repeat-report deduplication was not explicitly documented; Esan 2015 and McKenna 2025 retain some concerns because their specialist mixed-status service frames are not representative of a general criminal-justice population.

| Study | Q1 | Q2 | Q3 | Q4 | Q5 | Q6 | Q7 | Q8 | Q9 | Q10 | Q11 | Consensus |
|---|---|---|---|---|---|---|---|---|---|---|---|---|
| Tint 2017 | NA | NA | Y | Y | Y | N | U | Y | N | N | Y | Higher concern |
| Yu 2021 | Y | Y | Y | Y | Y | Y | Y | Y | Y | NA | Y | Lower concern |
| Mouridsen 2008 | Y | Y | Y | Y | Y | Y | Y | Y | Y | NA | Y | Lower concern |
| Mordre 2011 | Y | Y | Y | Y | Y | Y | Y | Y | Y | NA | Y | Lower concern |
| Siponen 2023 | Y | Y | Y | Y | Y | Y | Y | Y | Y | NA | Y | Lower concern |
| Maras 2024 | Y | Y | U | Y | Y | Y | U | Y | Y | Y | Y | Some concerns |
| Lundström 2014 | Y | Y | Y | Y | Y | Y | Y | Y | Y | NA | Y | Lower concern |
| Heeramun 2017 | Y | Y | Y | Y | Y | Y | Y | Y | Y | NA | Y | Lower concern |
| Yu 2019 | Y | Y | Y | Y | Y | Y | Y | Y | Y | Y | Y | Lower concern |
| Edberg 2022 | Y | Y | Y | Y | Y | Y | Y | Y | Y | Y | Y | Lower concern |

Table S3b note. Axis B used the design-appropriate JBI tool at the study-component level. Cohort/registry components used the revised JBI cohort checklist (Q1 group similarity/source population; Q2 exposure ascertainment; Q3 exposure measurement validity/reliability; Q4 confounders identified; Q5 strategies for confounding; Q6 outcome-free status at baseline where applicable; Q7 outcome measurement validity/reliability; Q8 follow-up time; Q9 completeness of follow-up; Q10 handling of incomplete follow-up; Q11 appropriate statistical analysis). Analytical cross-sectional components used the JBI analytical cross-sectional checklist and are marked/annotated according to the applicable domains rather than interpreted as an 11-item cohort score. NA denotes a non-applicable cohort item. Consensus categories follow the rule stated in Methods. Mordre 2011 is classified as lower concern because all applicable displayed domains are affirmative. Material evidence notes: Tint 2017 is higher concern because baseline/outcome ascertainment and attrition-related domains materially affect interpretation; Maras 2024 retains some concerns because sample/measurement uncertainties affect the selected estimate; registry cohorts with affirmative JBI items may still be downgraded in GRADE for indirectness, inconsistency, or imprecision.

## Supplementary Figure S1. JBI Traffic-Light Summary

A compact study-by-domain traffic-light display is provided below. Y = Yes; U = Unclear; N = No; NA = Not applicable. The detailed consensus decisions are authoritative; the visual is a display aid rather than a scoring system.

| Study | Axis | JBI item sequence | Consensus |
|---|---|---|---|
| Bukten 2024 | Axis A | Y Y Y Y Y Y Y Y Y | Some concerns |
| Murphy & Eley 2026 | Axis A | Y Y Y Y Y Y U Y Y | Some concerns |
| Billstedt 2017 | Axis A | Y Y Y Y Y Y Y Y Y | Lower concern |
| Sutton 2013 | Axis A | N N N Y N Y Y Y Y | Higher concern |
| Esan 2015 | Axis A | Y Y N Y Y Y Y Y Y | Some concerns |
| McKenna 2025 | Axis A | Y Y N Y Y Y Y U Y | Some concerns |
| Stinson & Robbins 2014 | Axis A | Y Y Y Y Y U U Y Y | Higher concern |
| Sitter 2025 | Axis A | Y Y Y Y Y Y Y Y Y | Some concerns |
| Eckert 2017 regular | Axis A | Y Y N Y Y Y Y Y Y | Some concerns |
| Eckert 2017 long-stay | Axis A | Y Y N Y Y Y Y Y U | Some concerns |
| Haw 2012 | Axis A | Y Y Y Y Y U Y Y Y | Some concerns |
| Dein 2021 | Axis A | Y Y Y Y Y N U Y Y | Higher concern |

| Study | Axis | JBI item sequence | Consensus |
|---|---|---|---|
| Helverschou 2015 | Axis A | Y Y Y Y Y Y Y Y Y | Lower concern |
| Boylu 2025 | Axis A | Y Y Y Y Y Y U Y Y | Some concerns |
| Edberg 2020 | Axis A | Y Y Y Y Y Y Y Y Y | Lower concern |
| Kumagami general 2009 | Axis A | N N Y Y N Y U Y U | Higher concern |
| Kumagami specialist 2009 | Axis A | N N N Y N Y U Y U | Higher concern |
| Rutten 2022 | Axis A | Y Y Y Y Y Y Y Y Y | Lower concern |
| Bath 2015 | Axis A | Y Y Y Y Y U Y Y Y | Some concerns |
| Siponmaa 2001 | Axis A | Y Y N Y Y U Y Y Y | Some concerns |
| Loizidou 2025 | Axis A | Y Y Y Y Y Y Y Y Y | Lower concern |
| Ross-Michaelides 2024 | Axis A | Y Y Y Y Y Y Y U Y | Some concerns |
| Tint 2017 | Axis B | NA NA Y Y Y N U Y N N Y | Higher concern |
| Yu 2021 | Axis B | Y Y Y Y Y Y Y Y Y NA Y | Lower concern |
| Mouridsen 2008 | Axis B | Y Y Y Y Y Y Y Y Y NA Y | Lower concern |
| Mordre 2011 | Axis B | Y Y Y Y Y Y Y Y Y NA Y | Some concerns |
| Siponen 2023 | Axis B | Y Y Y Y Y Y Y Y Y NA Y | Lower concern |
| Maras 2024 | Axis B | Y Y U Y Y Y U Y Y Y Y | Some concerns |
| Lundström 2014 | Axis B | Y Y Y Y Y Y Y Y Y NA Y | Lower concern |
| Heeramun 2017 | Axis B | Y Y Y Y Y Y Y Y Y NA Y | Lower concern |
| Yu 2019 | Axis B | Y Y Y Y Y Y Y Y Y Y Y | Lower concern |
| Edberg 2022 | Axis B | Y Y Y Y Y Y Y Y Y Y Y | Lower concern |

### Supplementary Table S4. Primary and Comparator Model Details

| Synthesis | Cohorts | Events/N | Primary GLMM, % [95% CI] | GLMM tau² | REML-mHK, % [95% CI] | Q (p) | I² |
|---|---|---|---|---|---|---|---|
| Forensic ID clinical services | Esan 2015; McKenna 2025 | 61/249 | 23.57 [10.67, 42.69] | .094 | 23.46 [0.26, 97.29] | 5.77 (.016) | 82.7% |
| General forensic responsibility evaluations (conditional sensitivity pool) | Helverschou 2015; Boylu 2025 | 119/15,235 | 0.89 [0.36, 2.23] | .126 | 0.91 [0.01, 53.95] | 16.15 (<.001) | 93.8% |
| Forensic youth referrals | Loizidou 2025; Ross-Michaelides 2024 | 181/770 | 23.51 [20.10, 27.08] | 0 | 23.51 [9.45, 47.51] | 0.10 (.748) | 0% |

Primary model: binomial GLMM with logit link and random intercept, maximum likelihood, 15-point adaptive Gauss-Hermite quadrature, and profile-likelihood confidence intervals. Comparator: inverse-variance logit random-effects model with REML tau² and modified Hartung-Knapp inference using a minimum variance multiplier of 1 and t(k-1). Q and I² refer only to the inverse-variance comparator. No continuity correction was used. Prediction intervals were not calculated because $k < 5$. Meta-regression, subgroup interaction tests, and funnel-asymmetry tests were not estimated because each compatible pool contained only two cohorts.

### Supplementary Table S5. Sensitivity and Source-Level Decisions

| Issue | Primary decision | Sensitivity / consequence |
|---|---|---|
| Norwegian forensic evaluation diagnoses | 45/3,382 currently confirmed diagnoses | Using all 48 historical diagnoses changes pooled general-evaluation estimate from 0.89% to 0.93% [0.35, 2.49]. |
| Turkish forensic evaluation report | Individual estimate is primary; pooled use is conditional on a unique-person denominator assumption | Because explicit repeat-report deduplication is not documented, the 0.89% two-cohort pool is presented only as an exploratory conditional sensitivity analysis; under the strict unique-person rule, Boylu is removed and Helverschou 45/3,382 (1.33%) remains the person-level estimate. |
| Sutton/Walters overlap | Sutton 22/37 retained; Walters not added | Walters alternative 27/43 is probably overlapping and was never combined with Sutton. |
| Murphy & Eley diagnostic definition | 39/319 confirmed diagnoses | Including suspected autism gives 123/319 and represents a different diagnostic construct. |

| Issue | Primary decision | Sensitivity / consequence |
|---|---|---|
| Eckert long-stay missing diagnosis | Observed 11/60 | Complete-sample scenarios: 11/61 and 12/61. |
| Maras cyber-offending model | Selected adjusted OR 0.94 [0.42, 2.14] | Earlier models: OR 1.13 [0.53, 2.41] and 1.04 [0.49, 2.22]. |
| Quadrature accuracy | 15 adaptive points | 61-point re-estimation reproduced displayed estimates and confidence limits; largest checked log-likelihood difference <1.3 x 10^-10. |
| Leave-one-out pooling | Not fitted when one cohort remains | Individual cohort estimates serve as the influence assessment; no one-study random-effects model was fitted. |

### Supplementary Table S6. Missing-Results and Source-Discrepancy Assessment

| Domain | Finding | Implication |
|---|---|---|
| Historical screening accounting | Exact title/abstract exclusion and retrieval-failure counts are not reconstructable from preserved logs. | No stage count was imputed; PRISMA figure reports the limitation explicitly. |
| Excluded-report reasons | 94 of 139 historical excluded report records lack a sufficiently documented specific primary exclusion reason. | Report-level total retained/excluded is used, but reasons are not retrospectively invented. |
| Routine clinical diagnosis | Several service cohorts depend on recorded diagnoses rather than active standardized case finding. | Potential under-ascertainment is considered in JBI/GRADE and interpretation. |
| Historical diagnostic categories | Some reports use PDD or Asperger-era classifications. | Historical constructs are retained with original labels and are not automatically equated with current ASD. |
| Registry windows | General prison registry cohorts use different observation windows. | Presented individually rather than combined as a common prevalence. |
| Assessment records | Rutten et al. provides an assessment-record fraction without established person-level deduplication. | Not pooled as person prevalence. |

| Domain | Finding | Implication |
|---|---|---|
| Outcome reporting | Axis B definitions, metrics, follow-up windows, and adjustment sets differ. | Endpoint-specific estimates are presented rather than a broad pooled effect. |
| Small-study effects | Only three compatible pools, each k = 2. | Funnel plots and asymmetry tests were not performed; absence of testing is not evidence of absence of reporting bias. |
| Search accounting | The 13,883-record working ledger is a preserved historical total; revision-stage six-source counts document later/expanded searches and are not arithmetically equivalent. | Figure 1 and SM1 distinguish the two accounting layers; no missing database-level crosswalk is reconstructed. |
| Search syntax | Exact verbatim historical database-specific strings/field settings were not preserved for every source. | Only verifiable archival details are reported; reconstructed syntax is not presented as contemporaneous search documentation. |
| Mixed-status forensic ID services | Esan and McKenna include mixed civil and criminal legal statuses. | Their synthesis is explicitly a specialist forensic-service caseload-composition estimate, not criminal-justice prevalence. |

## Supplementary Table S7. GRADE Evidence Profile

Two reviewers independently assessed certainty using the prespecified review adaptation of GRADE and resolved differences by consensus. For descriptive prevalence/event-frequency questions, observational evidence directly addressing the descriptive target was considered from a high-certainty starting point before downgrading. Comparative observational associations started at low certainty. Ratings concern confidence in magnitude and transferability, not whether autism or the reported outcomes exist in the relevant settings.

| Synthesis / outcome | Evidence | Risk of bias | Inconsistency | Indirectness | Imprecision | Missing results | Certainty |
|---|---|---|---|---|---|---|---|
| Specialist forensic ID services | 2 cohorts; 61/249; 23.57% [10.67, 42.69] | Serious | Serious | Not serious | Serious | Not assessable | Very low |
| General forensic responsibility evaluations | 2 cohorts; 119/15,235; 0.89% [0.36, 2.23] | Serious | Serious | Not serious | Serious | Not assessable | Very low |

| Synthesis / outcome | Evidence | Risk of bias | Inconsistency | Indirectness | Imprecision | Missing results | Certainty |
|---|---|---|---|---|---|---|---|
| Forensic youth referral services | 2 cohorts; 181/770; 23.51% [20.10, 27.08] | Not serious | Not serious | Serious | Serious | Not assessable | Low |
| Police interaction | 1 cohort; 46/284; 16.20% [12.37, 20.93] | Serious | Not assessable | Serious | Serious | Not assessable | Very low |
| Charge/conviction outcomes | 4 source studies; non-combinable: 5.28%; RR 0.50; HR 0.40; sex-specific HR 0.62/0.90 | Not serious to serious | Serious | Serious | Serious | Not assessable | Very low |
| Cyber offending | 1 cohort; OR 0.94 [0.42, 2.14] | Serious | Not assessable | Serious | Very serious | Not assessable | Very low |
| Violent conviction/criminality | 3 source studies; OR 1.10; RR 1.39 / 0.85 adjusted; sex-specific HR 1.19/2.87 | Not serious | Serious | Serious | Serious | Not assessable | Very low |

| Synthesis / outcome | Evidence | Risk of bias | Inconsistency | Indirectness | Imprecision | Missing results | Certainty |
|---|---|---|---|---|---|---|---|
| IPV arrest | 1 cohort; HR 0.70 [0.30, 1.60] | Not serious | Not assessable | Serious | Very serious | Not assessable | Very low |
| Reconviction after forensic assessment | 1 cohort; HR 0.60 [0.50, 0.80] | Not serious | Not assessable | Very serious | Not serious | Not assessable | Very low |

## Supplementary Note S1. Interpretation of the Revised Evidence Structure

The revised synthesis deliberately avoids a single overall prevalence estimate across prisons, secure forensic services, courts, responsibility evaluations, and youth referral pathways. These populations arise through different selection mechanisms and diagnostic opportunities. The three pooled estimates therefore answer narrow population-specific questions and should not be compared as though they were estimates from a common sampling frame. Likewise, Axis B does not yield a single summary measure of criminal-justice involvement: police contact, charge history, conviction, cyber offending, violence, IPV arrest, and reconviction are distinct outcomes measured with different designs and effect metrics.
